# Can artificial intelligence generate scientific discussion that passes peer review for publication in a high-impact orthopaedic journal?

**DOI:** 10.1007/s11845-025-03971-y

**Published:** 2025-06-12

**Authors:** Gerard A. Sheridan, Lisa C. Howard, Michael E. Neufeld, Tom R. Doyle, Andrew J. Hughes, Peter K. Sculco, David E. Beverland, Donald S. Garbuz, Bassam A. Masri

**Affiliations:** 1 University of British Columbia, Vancouver, BC Canada; 2 Hospital for Special Surgery, New York, NY USA; 3 Royal College of Surgeons in Ireland, Dublin, Ireland; 4 Musgrave Park Hospital, Belfast, UK

**Keywords:** AI, Artificial intelligence, Author, Chat GPT, Large language model, Peer review

## Abstract

**Background:**

There is huge interest in the use of artificial intelligence (AI) in the production and assessment of academic material; however, the role of AI remains unclear.

**Aim:**

The purpose of this study was to perform a reviewer-blinded assessment of the quality of scientific discussion generated by an advanced AI language model (ChatGPT-4, Open AI) and determine whether this could be recommended for high-impact journal publication.

**Methods:**

The introduction, methods and results sections of a recently published article from a high-impact journal were input into a current AI model. The AI application then produced a discussion and conclusion based on the provided text using a standardized prompt. Six experienced blinded reviewers scored all five sections of the hybrid article. A one-way analysis of variance (ANOVA) was used to assess significant differences between scores of each section. Reviewers recommended a decision regarding the suitability of the article for publication.

**Results:**

AI composed a scientific discussion and conclusion. The median score was 80 (IQR 70–90) for introduction, 77.5 (IQR 70–90) for methods, 82.5 (IQR 50–90) for results, 60 (IQR 40–75) for discussion and 60 (IQR 40–80) for the conclusion. The median scores for the AI-generated sections were non-significantly lower than other sections (*p* = 0.37). The majority of reviewers (5/6, 83%) recommended “acceptance for publication after major revision”. One reviewer recommended “resubmission with no guarantee of acceptance”. There were no recommendations for rejection.

**Conclusion:**

Current AI large language models are now capable of generating content that passes experienced peer review and is acceptable for publication in a high-impact orthopaedic journal, after revision. There are still many concerns regarding the integration of AI into the process of scientific writing, mainly the tendency of AI to rely on advanced pattern recognition and fabricated or inadequate references.

**Level of evidence:** Level IV

## Introduction

The role of artificial intelligence (AI) in medical research has been progressing at a significant rate in recent years. Large language models such as Chat GPT (Open AI, San Francisco, CA 94110, USA) have been shown to be capable of achieving the equivalent of a passing score for a third-year medical student on the United States Medical Licensing Examination (USMLE) [1]. As these large language models are evolving at an exponential rate, we are seeing improvements in their performance over very short periods of time, the latest of which can be seen with the release of ChatGPT-4 in March 2023. A study by Tagaki et al. demonstrated how GPT-4 outperformed GPT-3.5 in the Japanese Medical Licensing Examination (JMLE) [2]. GPT-4 was superior to GPT-3.5 in terms of accuracy and in answering difficult questions, and it also achieved the passing criteria for the JMLE, indicating that these large language models (LLMs) can match or outperform humans in knowledge-based medical fields already.

Within the orthopaedic community, the use of machine learning has already been described as a tool to assist in clinical decision making to improve patient care, for example, in predicting outcomes following Irrigation and Debridement (I&D) surgery for prosthetic joint infection (PJI) [3]. AI has also been shown to be capable of analysing radiographic images with significant precision in the context of hip and knee arthroplasty [4]. In addition to the role of AI in the clinical aspects of orthopaedic patient care, AI has also integrated with the orthopaedic research community. It is becoming a tool used appropriately and inappropriately in the production of orthopaedic research and in the generation of scientific content, similar to a human author.

In 1950, Alan Turing described the Turing Test (i.e. Imitation Game) where a machine passes the test provided its responses were indistinguishable from a human [5]. It has recently been shown that reviewers struggle to identify AI generated abstracts, highlighting the progress LLMs have made [6]. The role of LLMs in the production of scientific content is still unclear, and the scientific community is attempting to quickly implement safeguards in the interest of upholding scientific integrity and the quality of scientific content. The Editors-in-Chief for *The Bone & Joint Journal*, *The Journal of Bone & Joint Surgery, Clinical Orthopaedics and Related Research* and *Journal of Orthopaedic Research* published recommendations regarding the involvement of AI in the generation of scientific articles [7]. The recommendations include “AI applications cannot be listed as authors. Whether and how AI applications were used in the research or the reporting of its findings must be described in detail in the Methods section and should be mentioned again in the Acknowledgements section”.

The purpose of this study was to perform a reviewer-blinded assessment of the scientific quality of a discussion and conclusion section generated by an advanced AI LLM, and ultimately determined whether AI-generated content would be recommended for peer-reviewed publication. The hypothesis was that the discussion would pass peer review and would not be identified as computer-generated.

## Methods

### AI generation of discussion and conclusion

On May 10, 2023, a PubMed search was performed to identify recently published articles in the field of hip arthroplasty published in a Q1 high impact orthopaedic journal as identified in 2022 Journal Impact Factor, Journal Citation Reports (*Clarivate, 2023*). The article entitled “Survival of the Exeter V40 short revision (44/00/125) stem when used in primary total hip arthroplasty” published in *Bone & Joint Journal* on May 1, 2023, was arbitrarily selected [8]. The manuscript was reviewed in full (G.A.S & T.R.D). Following this, the introduction, methods and results sections from the article were inserted into the ChatGPT-4 application along with the references cited in the original discussion of the article [9–18] using the following specific instructions:

“Referencing all citations listed in the reference list, write a discussion section (1500 words total) and conclusion to follow the introduction, methods and results section listed here. This discussion and conclusion should be composed in a style that would be suitable for publication in the Bone & Joint Journal (BJJ). Reference list (Include all citations listed here in your discussion):”

The scientific discussion and conclusion generated by ChatGPT-4 were then copied directly, without any form of human editing, into a hybrid document to replace the original discussion and conclusion. This new hybrid document included the original introduction, methods and results section with the addition of the new AI-generated discussion and conclusion sections.

### Reviewer selection process

Six fellowship-trained arthroplasty surgeons who work in large academic hospitals with experience in reviewing articles on the topic of total hip arthroplasty for high-impact orthopaedic journals were recruited to participate in the study (LCH, MEN, PKS, DEB, DSG, BAM). The reviewers were recruited from three different countries, in North America and Europe. The years of experience of Q1 peer review for each reviewer are summarized in Table 1.

**Table 1:** Peer reviewer academic experience

Reviewer	Journals	Impact factor (June 2023)	Experience (years)
1	*Journal of Bone and Joint Surgery* *Clinical Orthopaedics and Related Research* *Journal of Arthroplasty*	6.558 4.837 4.435	> 20 years
2	*Bone & Joint Journal* *Clinical Orthopaedics and Related Research* *Hip International*	5.385 4.837 1.756	> 25 years
3	*Bone & Joint Journal* *Journal of Arthroplasty* *BMC Musculoskeletal Disorders*	5.385 4.435 2.32	7
4	*Journal of Bone and Joint Surgery*	6.558	4
5	*Bone & Joint Journal* *Journal of Arthroplasty* *Bone & Joint Open*	5.385 4.435 2.8	4
6	*American Journal of Sports Medicine* *Hip International* *Injury*	6.057 1.756 2.687	3

All reviewers confirmed that they had not read the full article of the circulated manuscript and were unaware of its authorship. All reviewers had no affiliation to the article authors’ institution. All reviewers were blinded to the methodology of the current study and, as such, were completely unaware that part of the document they were reviewing was generated by AI.

### Reviewer scoring

The hybrid document was then circulated to all reviewers with an accompanying reviewer score sheet (Appendix). The scoring system involved numerical grading of each individual section of the article (maximum score of 100 for each section). The reviewers were also asked to give one of the following overall decisions: (1) *Accept*, (2) *Accept with minor revisions*, (3) *Accept after major revisions*, (4) *Do not accept yet (authors may resubmit with no guarantee of acceptance)*, (5) *Reject.* All score sheets were returned to the corresponding author and analysed.

### Statistical analysis

Data distribution was assessed using the Shapiro–Wilk test, which confirmed that the data was not normally distributed. Therefore, scores were expressed using median values and inter-quartile ranges (IQR). Boxplot graphs were generated to demonstrate median values and IQRs. A one-way analysis of variance (ANOVA) was then performed to assess any significant difference between the five median scores. A *p*-value of < 0.05 was taken to be statistically significant. All statistical analyses were performed using Stata/IC 13.1 for Mac (64-bit Intel) (College Station, TX, 77,845). Institutional Review Board (IRB) approval was not required given the nature of the study.

## Results

**Fig. 1:**
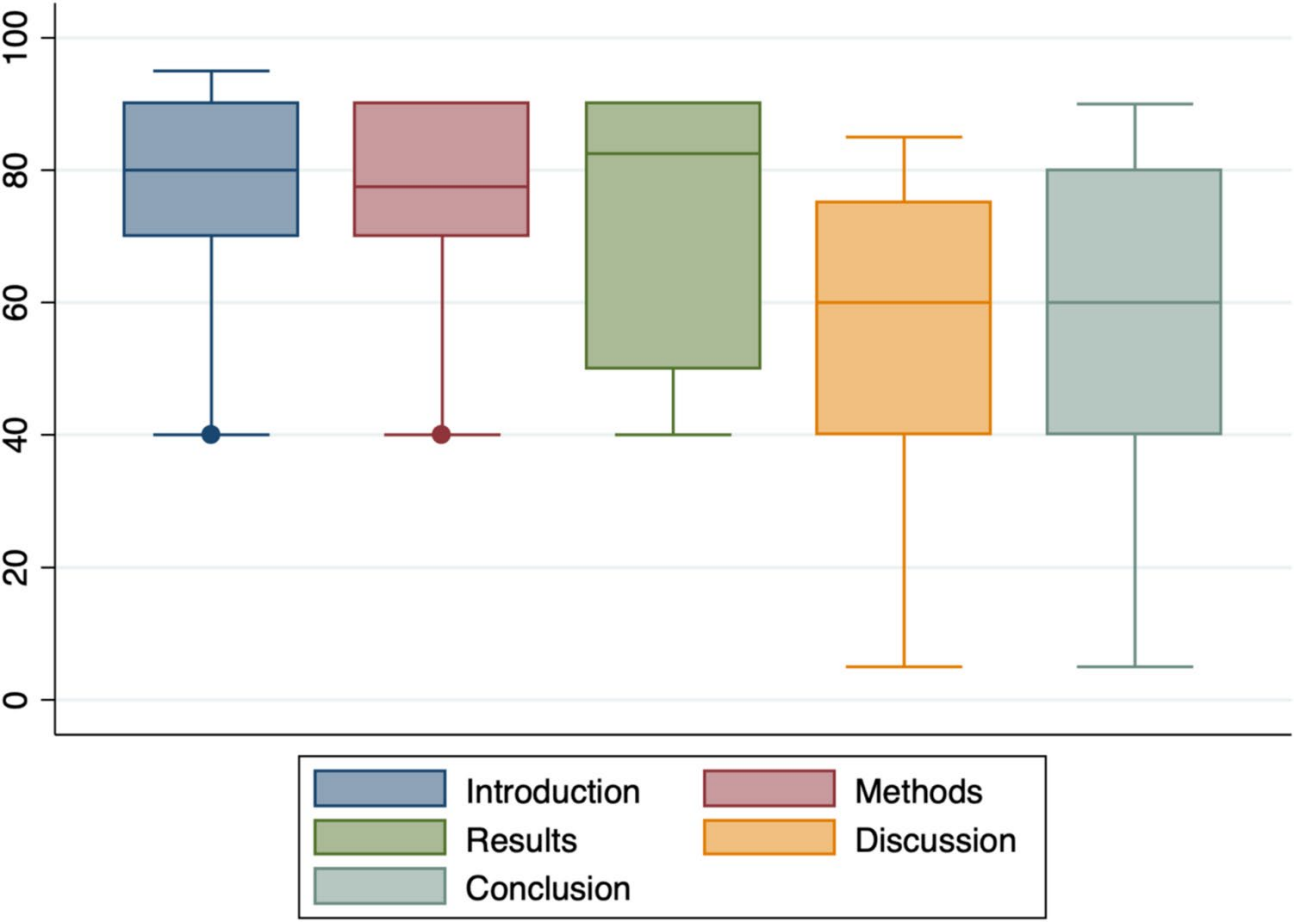
Boxplot of academic quality scoring (0–100)

The individual reviewer scores for each section and overall decision are detailed in Table 2. The median score was 80.0 (IQR 70–90) for introduction, 77.5 (IQR 70–90) for methods, 82.5 (IQR 50–90) for results, 60.0 (IQR 40–75) for discussion and 60.0 (IQR 40–80) for the conclusion. There was no significant difference in the median scores for the discussion and conclusion compared to other sections, as shown in Fig. 1 (*p* = 0.37). No reviewer raised concerns in their free text comments regarding the potential use of AI in the manuscript.

**Table 2:** Individual reviewer scores and overall decision

Reviewer	Section	Score (100 max)	Overall decision
1	- Introduction - Methods - Results - Discussion - Conclusion	80 70 90 60 50	*Accept after major revisions*
2	- Introduction - Methods - Results - Discussion - Conclusion	40 40 40 40 40	*Accept after major revisions*
3	- Introduction - Methods - Results - Discussion - Conclusion	80 90 90 85 90	*Accept after major revisions*
4	- Introduction - Methods - Results - Discussion - Conclusion	90 70 80 75 80	*Accept after major revisions*
5	- Introduction - Methods - Results - Discussion - Conclusion	95 85 85 5 5	*Do not accept yet (authors may resubmit with no guarantee of acceptance)*
6	- Introduction - Methods - Results - Discussion - Conclusion	70 90 50 60 70	*Accept after major revisions*

There were no recommendations suggesting rejection of the manuscript, with all inviting edits and resubmission. The majority of reviewers (5/6, 83%) recommended “Accept after major revisions”. One reviewer recommended “Do not accept yet (authors may resubmit with no guarantee of acceptance)”. This reviewer noted in their free text comments “Further discussion of limitations, what the study actually showed, and comparison to pertinent literature is required. Since there is limited published evidence regarding the use of this stem in primary settings, the authors should state this as well. Needs to discuss patient selection problems with the stem”.

## Discussion

The purpose of the current study was to assess the extent to which AI generated scientific writing would be able to withstand the scrutiny of experienced high-impact factor orthopaedic peer review. Based on the current results, AI was able to compose a nuanced scientific discussion and conclusion based on the introduction, methods and results sections. While there was a reduction in the median scores for discussion and conclusion, this difference was not statistically significant. Furthermore, the reviewers did not identify the use of AI or express criticism which warranted rejection of the manuscript. On the contrary, five of the six reviewers recommended publication after revisions, a verdict which will often proceed to manuscript publication. Hence, it was demonstrated that AI can generate scientific content that experienced reviewers identify as suitable for publication in Q1 orthopaedic journals.

Regarding the lower scores for the discussion and conclusion sections, it should be noted that at the time of the review of the hybrid manuscript, the introduction, methods, and results had already undergone peer review in the *Bone & Joint Journal* with subsequent revisions prior to publication in its final format. In contrast, the discussion and conclusion sections generated by AI were taken directly from the LLM without any human editing. Therefore, the human-generated sections had a distinct advantage having already undergone peer review and revision, while the AI sections had not. In a similar vein, the LLM was provided citations from the original article to assist the writing of the discussion, which likely had an impact on the quality of its output, as LLM task performance is known to be related to the prompts it is provided with [19].

If AI applications are to be involved in the process of scientific manuscript generation in the future, the time taken to edit an AI-generated discussion may be significantly lower than the time taken for a human to write the discussion section in its entirety. This time efficiency demonstrates one way in which AI may be able to improve the productivity of orthopaedic researchers in the future. However, concerning aspects of this method of content generation is the tendency of Chat-GPT to insert references throughout the body of text which are often fabricated, from low quality or non-peer-reviewed sources [19]. The generated discussion was instructed to include all references from the original article and did so with the exception of Hamilton et al. [18], producing a viable scientific discussion that was deemed to be coherent by expert review. However, this shows imperfect execution of the instructions and highlights a potential pitfall with LLM writing. The AI model used in this study did not have real-time internet access to the full articles for the references provided, nor does it have access to articles published after its data training set was compiled. So while the generated content may appear coherent and accurate, the model is generating a discussion without access to the latest evidence and inserting references where it predicts they should be inserted. This is a limitation of AI written discussions as they may not be able to properly assess the nuanced risks vs. benefits of a treatment or the context specific application of the findings. The limitations of the training dataset may prevent the model writing coherently about new advances which is has not encountered before. This was born out in the comments of reviewer 5 who highlighted that there is limited published evidence on this topic and they felt the discussion inadequately addressed this. This may suggest the LLM struggled with a novel topic. Newer versions of Chat-GPT will have real-time internet access and may be able to avoid some of these issues, but currently, this is a real and valid concern. However, despite this, the hybrid document passed the scrutiny of experienced peer reviewers asked to judge it to the standards of a Q1 publication.

Irrespective of any advantages of AI involvement in scientific writing, the question remains whether the involvement of AI is the appropriate direction to take and to what extent it should be involved in the future. Polisetty et al. addressed the pitfalls associated with the use of AI in hip and knee arthroplasty research, quoting the following issues: “vernacular conflation, repackaging limited registry data, prematurely releasing internally validated prediction models, appraising model architecture instead of inputted data, withholding code, and evaluating studies using antiquated regression-based guidelines” [20]. The appraisal of model architecture instead of inputted data is especially relevant when using AI to generate scientific discussion. Even though the discussion may appear to be coherent and appropriately referenced, there is a risk that the results are not comprehended by the LLM. The model is essentially using pattern recognition from its training data to create a discussion rather than deeply considering the implications of the results. It has previously been shown that experienced reviewers struggle to identify AI-generated scientific abstracts [6]. However, the present study demonstrates a further development in LLM capabilities as much greater complexity is contained in the discussion section compared to an abstract. The fact that this discussion may be the product of an entity without any “higher-order understanding” is an issue that needs further exploration, as it seems based on the current study that “advanced pattern recognition” may pass as a substitute for “intelligent composition” from the perspective of a human reviewer.

In addition to problems with data interpretation and comprehension, there may also be problems at a more foundational level with the data itself, regardless of whether the AI comprehension is accurate or not. Odouye et al. discuss the fact that large language models are limited by the data that are provided to them [21]. For example, if there is any biased or inaccurate data present in a dataset for analysis, this bias or inaccuracy may be reinforced by the algorithm since machine learning algorithms can only be as objective as the data they are trained on. Therefore, the scientific community must be ever-diligent regarding the quality of data being input for analysis. The capability and advancement of AI technology will never be able to compensate for poor data quality.

This current study appears to be the first to assess the quality of AI-generated discussion as determined by blinded experienced reviewers of high-impact orthopaedic journals. There was no significant difference in the appraised quality of the discussion and conclusion sections than the other human-generated sections. The article was flagged for revisions; however, this is standard practice for a vast majority of submissions to Q1 journals. The major concern is that AI-generated content may be masquerading as intelligently composed content when, in actuality, it is simply an excellent example of pattern recognition.

### Limitations

It may be considered a limitation that introduction, methods and results had already undergone expert peer review and subsequent revision prior to the blinded review for this study, while the discussion section had not. Other potential limitations include the relatively small sample size of reviewers, the heterogeneity in the human peer review process and the potential biases of the reviewers.

## Conclusion

Current AI large language models are now capable of generating content that passes experienced peer review and is acceptable for publication in a high-impact orthopaedic journal, after revision. There are still many concerns regarding the integration of AI into the process of scientific writing, mainly the tendency of AI to rely on advanced pattern recognition and fabricated or inadequate references.
